# PTX3 acts as an extrinsic oncosuppressor

**DOI:** 10.18632/oncotarget.4845

**Published:** 2015-07-11

**Authors:** Eduardo Bonavita, Alberto Mantovani, Cecilia Garlanda

**Affiliations:** Humanitas Clinical and Research Center, Rozzano, Milan, Italy

**Keywords:** Immunology and Microbiology Section, Immune response, Immunity

Inflammation is an essential component of the tumor microenvironment that sustains tumor development and growth [1]. The role in cancer-related inflammation of innate immunity cells recruited in the tumor has been clarified in preclinical models. In contrast, the role of the humoral arm of the innate immune system, which includes biochemically heterogeneous molecules such as Complement components, collectins, ficolins and pentraxins, is still under investigated. The long pentraxin PTX3 represents a functional paradigm of humoral innate immunity [2]. By interacting with selected microbial moieties and playing opsonic activity via Fcγ receptors, and activating and regulating the Complement cascade, PTX3 acts as a functional ancestor of antibodies. PTX3 plays non-redundant roles in resistance against selected microbial pathogens and in regulating inflammatory and tissue repair responses [2, 3]. PTX3 is highly conserved in evolution and genetic evidence is consistent with a role of PTX3 in antimicrobial resistance in humans [3, 4].

We have recently investigated the role of the humoral arm of innate immunity in cancer-related inflammation using the long pentraxin PTX3 as a paradigm [5]. We found that PTX3-deficiency in mice caused increased susceptibility to mesenchymal and epithelial carcinogenesis in the models of 3-Methylcholanthrene (3-MCA)-induced carcinogenesis, and 7,12-dimethylbenz [α] anthracene/terephthalic acid (DMBA/TPA)-induced skin carcinogenesis. In these models, infiltrating leukocytes, in particular cells of the monocyte-macrophage lineage, and endothelial cells were a major source of PTX3 in response to locally produced IL-1, and both contributed to PTX3-dependent protection against carcinogenesis. PTX3-deficiency was associated with enhanced macrophage tumor infiltration, pro-inflammatory cytokine production, angiogenesis, complement C3 deposition and C5a levels, suggesting exacerbated cancer-related inflammation, whereas PTX3 had no direct effect on tumor cell proliferation. We further showed that genetic inactivation of C3 reverted the increased susceptibility to 3-MCA-induced carcinogenesis and macrophage recruitment and demonstrated that PTX3 regulated C3-deposition on sarcoma cells by interacting with and recruiting the negative regulator Factor H. In addition, CCL2-inibition was sufficient to revert the increased susceptibility of PTX3-deficent mice to 3-MCA and the M2-like phenotype of tumor-associated macrophages.

We also showed that PTX3-deficiency was associated to increased DNA damage, as demonstrated by increased *Trp53* mutations, oxidative DNA damage and expression of DNA damage (DDR) markers, in line with the hypothesis that cancer-associate inflammation contributes to genetic events that cause cancer and to the genetic instability of tumors. We finally showed that *PTX3* promoter and regulatory regions were highly methylated in selected human mesenchymal and epithelial tumors, in contrast to the normal counterpart. In particular, in colorectal cancer, *PTX3* epigenetic modifications occurred early in progression already at the level of adenomas. *PTX3* methylation was responsible of silencing of PTX3 protein expression. Indeed, treatment of colorectal cancer cells with a methylation inhibitor (5-Aza-2′-deoxycytidine) was sufficient to restore the histone modifications associated to transcriptional activation and the interaction of transcription factors responsible of PTX3 expression (e.g. NF-kB, c-Jun, c-Fos) with their binding sites in the *PTX3* promoter region, and rescued PTX3 protein expression in response to an inflammatory stimulus.

**Figure 1:**
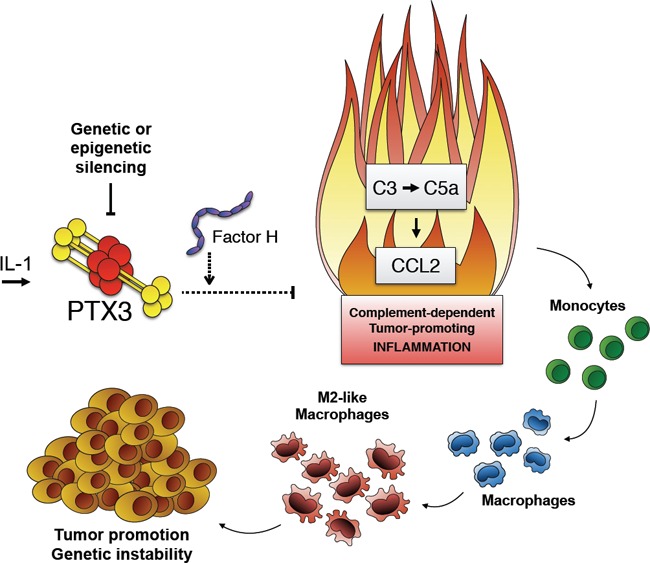
Schematic view of the mechanisms involved in the regulatory role played by PTX3 in cancer-related inflammation Either genetic or epigenetic silencing of PTX3 trigger Complement-dependent tumor-promoting inflammation and favor macrophage accumulation, tumor growth and gene instability.

Several observations support Complement-mediated recognition of malignant cells and Complement activation in many cancers. However, no formal evidence supports the existence of an effective immune surveillance mediated by Complement during carcinogenesis. On the other side, Complement is not considered a canonical component of tumor promoting inflammation [1], even if C5a has been shown to play a pro-tumoral role by recruiting myeloid-derived suppressor cells, amplifying their T cell suppression activity and CCL-2 production [6]. In our study we found that C3-deficiency was associated to decreased susceptibility to mesenchymal and epithelial carcinogenesis. Chemoattractants including C5a induce chemokines. Thus, in 3-MCA-induced sarcoma, unleashed Complement activation and increased C5a production associated to PTX3-decifiency are likely responsible of exacerbated production of CCL2, which in turn recruits tumor promoting macrophages in increased numbers and favours M2-like polarization [1] (Figure 1). In agreement with our results, a recent study showed that deficiency in key components of complement (e.g., C3, C5, or C5a receptor) was associated to resistance to colitis-associated colon cancer induced by azoxymethane and dextran sulfate sodium in mice. In this model, C5a represented a potent inducer of IL-1β in neutrophil that promoted colon carcinogenesis by eliciting IL-17 response in intestinal myeloid cells [7]. These and our results demonstrate that Complement is an essential component of tumor promoting inflammation in specific cancers. In a previous study of HPV-driven squamous cell skin carcinogenesis, C3-deficiency had no impact on cancer formation and progression [8]. Therefore, the role of Complement may well depend upon the tissue context and driving molecular pathways.

Thus, our study show that an essential component of the humoral arm of innate immunity and regulator of Complement activation acts as an extrinsic oncosuppressor gene in mouse and man by acting at the level of Complement-mediated, macrophage-sustained, tumor promoting inflammation (Figure 1).

These observations provide the first genetic evidence that an effector molecule of humoral innate immunity can act as an extrinsic oncosuppressor gene and introduce Complement as a key component of tumorpromoting cancer-related inflammation. These results call for a reassessment of the role of Complement in different human tumors.
